# Gradual collapse of nuclear wave functions regulated by frequency tuned X-ray scattering

**DOI:** 10.1038/srep43891

**Published:** 2017-03-07

**Authors:** Nina Ignatova, Vinícius V. Cruz, Rafael C. Couto, Emelie Ertan, Andrey Zimin, Freddy F. Guimarães, Sergey Polyutov, Hans Ågren, Victor Kimberg, Michael Odelius, Faris Gel’mukhanov

**Affiliations:** 1 Theoretical Chemistry and Biology, Royal Institute of Technology, S-10691, Stockholm, Sweden; 2 Institute of Nanotechnology, Spectroscopy and Quantum Chemistry, Siberian Federal University, 660041, Krasnoyarsk, Russia; 3 Instituto de Química, Universidade Federal de Goiás, Campus Samambaia, CP 131 CEP 74001-970, Goiânia-GO, Brazil; 4 Department of Physics, Stockholm University, AlbaNova University Center, 10691, Stockholm, Sweden

## Abstract

As is well established, the symmetry breaking by isotope substitution in the water molecule results in localisation of the vibrations along one of the two bonds in the ground state. In this study we find that this localisation may be broken in excited electronic states. Contrary to the ground state, the stretching vibrations of HDO are delocalised in the bound 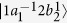 core-excited state in spite of the mass difference between hydrogen and deuterium. The reason for this effect can be traced to the narrow “canyon-like” shape of the potential of the 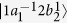 state along the symmetric stretching mode, which dominates over the localisation mass-difference effect. In contrast, the localisation of nuclear motion to one of the HDO bonds is preserved in the dissociative core-excited state 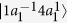. The dynamics of the delocalisation of nuclear motion in these core-excited states is studied using resonant inelastic X-ray scattering of the vibrationally excited HDO molecule. The results shed light on the process of a wave function collapse. After core-excitation into the 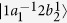 state of HDO the initial wave packet collapses gradually, rather than instantaneously, to a single vibrational eigenstate.

The phenomena of localisation and delocalisation become increasingly important in studies related to migration of vibrational excitations in solid matter and liquids. Philip Anderson discovered that the addition of a critical amount of disorder to a periodic system results in the interference of multiply scattered electrons, which can cause their wave functions to localise while the electrons come to an effective standstill state^1^. Recently it was recognised that vibrational modes^2^ and surface plasmons^3^ of disordered systems are not universally localised according to Andersons mechanism and have properties both of localisation and delocalisation. Special attention has been paid to the confinement of optical phonons in novel kinds of heterostructures and isotopic superlattices^4^. The localisation of vibrational modes is of crucial importance also in molecules, especially from the point of view of migration of vibrational excitations in large systems or in liquids^5^.

The interest in vibrational mode localisation is stimulated by studies of bond-selective vibrationally mediated photochemistry^6^,^7^,^8^,^9^. The isotopomer of the water molecule, HDO, plays a central role in such studies^10^,^11^,^12^,^13^,^14^. In infra-red (IR) spectroscopy the diluted isotope substitution of water, HDO, has been widely used as a unique probe of local hydrogen bond environments and of reorientational motion and hydrogen bond dynamics using pump-probe IR and multidimensional correlation techniques^15^,^16^,^17^. The symmetric character of the ordinary water molecule is reflected by its vibrational wave functions, with the two O-H bonds vibrating coherently forming the symmetric and antisymmetric stretching modes delocalised on both O-H bonds, leading to the properties observed previously in resonant inelastic X-ray Raman scattering (RIXS)^18^,^19^ and IR studies^20^,^21^,^22^,^23^. However, as soon as this symmetry is broken, as in HDO where one hydrogen atom is substituted by deuterium, the coherence of the O-H and O-D stretching vibrations is destroyed and the vibrational motion in the ground electronic state becomes localised to one of the bonds. Even though this localisation holds true for the ground state vibrations in HDO and is well established by numerous experiments^10^,^11^,^12^,^13^,^14^, it does not represent a strict physical principle.

We show in this article that even though the ground state vibrations of HDO are fully localised to the bonds, the vibrations become delocalised in certain core-excited states (Fig. 1). The reason for this unexpected delocalisation of vibrations in the asymmetric isotopomer HDO can be found in the competition between the symmetric shape of the potential energy surface (PES) and the asymmetric kinetic energy operator, which allows for a coexistence of localised and delocalised nuclear motions in the same molecule. We show that the mode delocalisation in an excited electronic state, exemplified by the HDO molecule, can be controlled by using an IR pump-RIXS probe technique^24^,^25^. Employing a pump IR pulse one can selectively populate a particular localised vibrational level of the ground electronic state, for example the *ψ*_1,0_ state localised in the O-D bond, Fig. 1a. Then a probe X-ray photon promotes the system into a core-excited state, where the nuclear motion may be localised along the selected O-D bond in the case of the dissociative 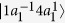 state (left panel of Fig. 1b) and delocalised in the case of the bound 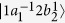 state (right panel in Fig. 1b). Subsequent decay back into the ground electronic state will populate vibrations localised either only on the selected O-D bond or on both O-D and O-H bonds, depending on the degree of delocalisation in the core-excited state. The final population of the ground state vibrational modes, which are localised on different bonds, gives direct information about the degree of delocalisation in the core-excited state. One should notice that in the H_2_O molecule the delocalised character of the ground state vibrational wave functions is preserved in the vibrational functions of the investigated core-excited states (Fig. 1b).

## Results and Discussion

### Mechanism of delocalisation of vibrational excitations in asymmetric molecules

The HDO molecule is an asymmetric isotopomer of H_2_O with the symmetry reduced from C_2*v*_ to C_*s*_. In spite of the fact that the electronic structure of HDO is equivalent to H_2_O, the nuclear dynamics along the O-H and O-D bonds is asymmetric due to the mass difference (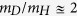) which introduces an asymmetry in the nuclear Hamiltonian of the stretching motion via the kinetic energy operator *K* (see Methods). The localisation or delocalisation of the vibrations in HDO thus depends on the competition between the symmetric potential (*U*_*i*_(*R*_1_, *R*_2_) = *U*_*i*_(*R*_2_, *R*_1_)) and the asymmetric kinetic energy operator *K* responsible for the dynamics, where *R*_1_ and *R*_2_ are the lengths of the O-H and O-D bonds. While the symmetric potential, being the same for H_2_O and HDO, forces symmetry preservation and maintains the vibrations delocalised on both bonds of the HDO molecule, the role of the asymmetric kinetic energy operator is qualitatively different: The mass difference (*m*_*H*_ ≠ *m*_*D*_) results in a localisation of the vibration on one bond. It is here instructive to look at the spatial shape of the nuclear wave functions, which directly indicates if the vibration is localised on one bond or it is delocalised over the both bonds (see Fig. 2 and Supplementary Fig. S1). The vibrationally (IR) excited *ψ*_1,0_ and *ψ*_0,1_ wave functions of the ground state HDO are strongly localised along either the O-D bond or the O-H bond, whereas in H_2_O the corresponding wave functions are delocalised. This motivates us to assign the vibrational states 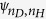 of HDO in the electronic ground state using the quantum numbers *n*_*D*_ and *n*_*H*_ of the vibrational states localised to the O-D and O-H bonds, respectively. In contrast, the vibrational states 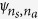 of the symmetric H_2_O molecule are fully delocalised in all electronic states. We use for this molecule the quantum numbers *n*_*s*_ and *n*_*a*_ of coupled symmetric and asymmetric stretching normal modes, respectively.

**Figure 1:**
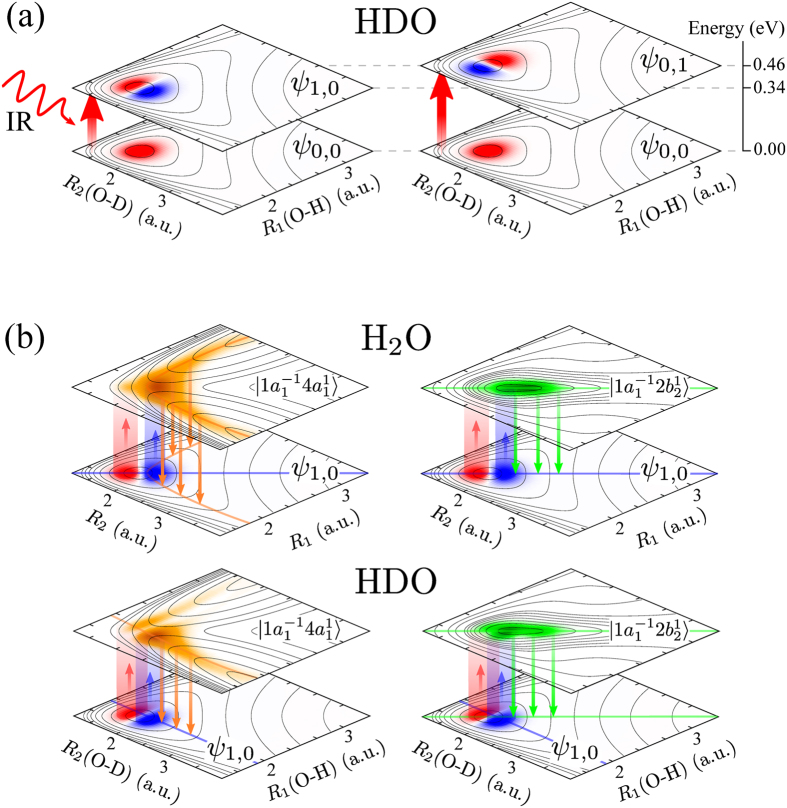
Localisation/delocalisation scheme of the IR-pump RIXS-probe for the H_2_O and HDO molecules. (**a**) Scheme of the IR pump process (in the ground electronic state) depicted for the HDO molecule. (**b**) Scheme of the RIXS probe process via the core-excited states 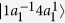 and 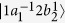. In H_2_O (upper panel), the delocalised initial state *ψ*_1,0_ leads to a delocalised core-excited wave packets |Ψ(∞)|^2^ (see Eq. 5), in both core-excited states. In HDO (lower panel), the initial localised wave function leads to a localised wave packet in the dissociative 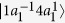 state and a delocalised one in the bound 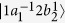 core-exited state. The contour lines represent the potential energy surface of the ground and core-excited states.

**Figure 2:**
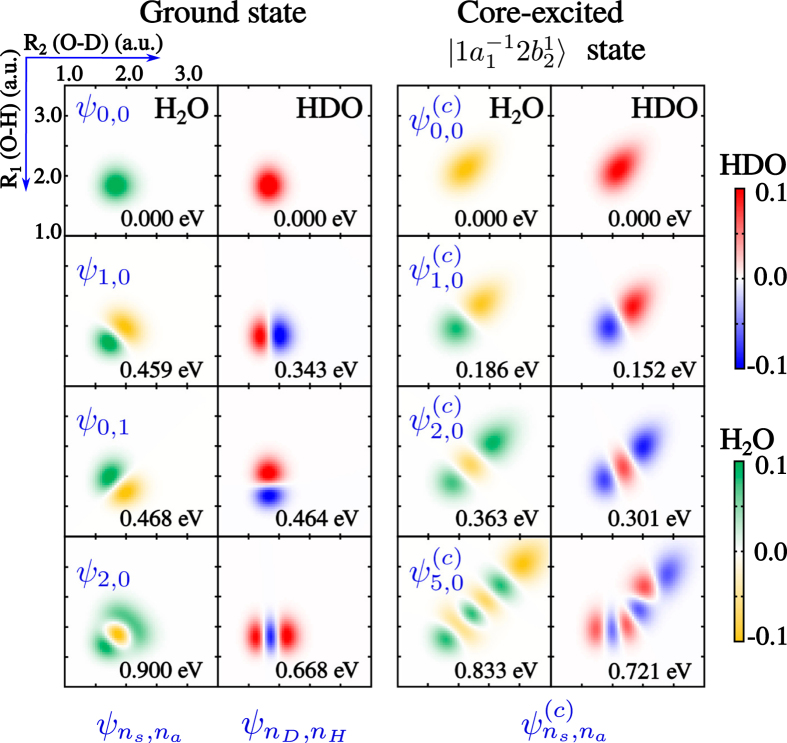
Vibrational wave functions of H_2_O and HDO for the ground and core-excited 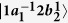 states. The energy of each vibrational state, with respect to zero-point energy, is shown inside each subpanel. (See Supplementary Fig. S1 for more vibrational states.)

In the HDO molecule, the difference between the ground and 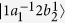 core-excited state wave functions is remarkable (see Fig. 2 and Supplementary Fig. S1). In spite of the strong asymmetry of HDO, the few lowest stretching vibrations in the core-excited state are almost fully delocalised, very similar to the symmetric H_2_O molecule. The main reason for this effect is that the narrow “canyon-like” PES of the bound 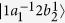 core-excited state is aligned along the symmetric stretching coordinate between the bonds (Fig. 1), which traps the O-H and O-D vibrations, thereby, overcoming the localisation trend imposed by the asymmetric kinetic energy operator (see Methods Eq. (17)). Due to the delocalisation of the vibrations in the 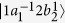 core-excited state, we use for this state the same assignment of vibrations 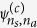 as in H_2_O. To avoid confusion, we mark the vibrational states of the core-excited state by the label (*c*). For further clarity, the notations used for ground and core-excited vibrational states can be summarised as follows


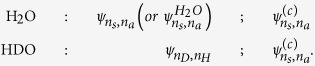


Using perturbation theory we can now quantify the degree of the localisation of the vibrational states in HDO by means of the kinetic energy operator of H_2_O and the asymmetric perturbation *δK*, related to the mass difference of H and D


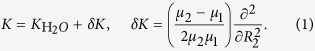


Here *μ*_1_ = *m*_*H*_*m*_*O*_/(*m*_*H*_ + *m*_*O*_) and *μ*_2_ = *m*_*D*_*m*_*O*_/(*m*_*D*_ + *m*_*O*_) are the reduced masses. The *ab initio* vibrational wave functions shown in Fig. 2 provide direct information about the localisation or delocalisation of the stretching vibrations. In order to explain the physical mechanism of localisation/delocalisation and the shape of the wave functions, shown in Fig. 2, let us consider a simple two-level model which catches the essence of the investigated effect. Apparently, to get the localisation we should mix the symmetric and antisymmetric wave functions. For HDO these can be constructed from the symmetric 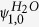 and antisymmetric 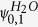 wave functions of H_2_O (Fig. 2), which are mixed by the perturbation *δK*


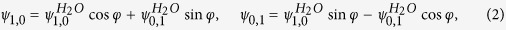


where 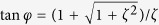. The degree of localisation is defined by the dimensionless parameter


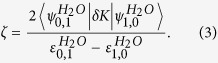


Apparently, the spacing 
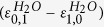
 between vibrational levels is directly related to the shape of the potential. In the ground state H_2_O, the symmetric and antisymmetric vibrationally excited modes are almost degenerate, 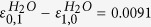 eV (see eigenvalues in Fig. 2), which makes the parameter |*ζ*| large. This explains the perfect localisation of vibrational states (2) in the ground state HDO, (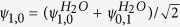, 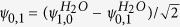, 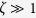) as seen in Fig. 2. These wave functions correspond to the HDO stretching vibrations localised on either the O-D bond or the O-H bond. The picture drastically changes for the 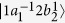 core-excited state where the PES shape resembles the narrow “canyon” along the symmetric stretching coordinate (Fig. 1). In this case, the spacing between the 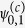 and 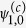 states of H_2_O is large (
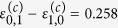
 eV (see eigenvalues in Fig. 2 and Supplementary Fig. S1), and hence, the parameter *ζ* is small. Contrary to the ground state, the vibrational wave functions of the asymmetric HDO molecule are delocalised, almost identically to the symmetric H_2_O wave functions.

A natural method to study localisation/delocalisation of vibrational excitations in the core-excited state of HDO is the pump-probe RIXS technique, in which an IR laser is used to pump the system to a vibrational level localised along either the O-H bond or the O-D bond. In order to find “experimental” evidence of the delocalisation of stretching vibrations in the 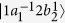 core-excited state, the vibrationally excited HDO molecule is probed by RIXS starting from different initial vibrational states: *ψ*_0,0_ (delocalised), *ψ*_1,0_ (localised along the O-D bond) and *ψ*_0,1_ (localised along O-H bond) (see Fig. 2). As we show below, the X-ray scattering back into the ground electronic state provides direct means to quantify the degree of delocalisation of the vibrational excitation in the 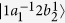 core-excited state.

In the present study, we focus on quasi-elastic RIXS channels, namely, the scattering via the two lowest core-excited states back into the ground electronic state. As we have shown recently^26^,^27^, the main spectral features of the RIXS spectrum in this case originate from the two stretching vibrational modes, which are strongly coupled. Following this result, we neglect the bending normal mode excitation in our simulations, while treating explicitly the coupled nuclear dynamics in the stretching modes (Fig. 1) using the strict 2D Hamiltonian (see Methods Eq. (17)) in a time-dependent representation of the Kramer’s-Heisenberg formalism (see Supplementary Material for details). The RIXS processes is sensitive to the nuclear dynamics in the core-excited state, defined by the nuclear wave packet


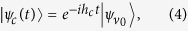


where *ν*_0_ = (*n*_*s*_, *n*_*a*_) (*ν*_0_ = (*n*_*D*_, *n*_*H*_)) is the initial vibrational level of the electronic ground state of H_2_O (HDO) with energy 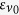. The RIXS cross section is computed as^28^,^29^


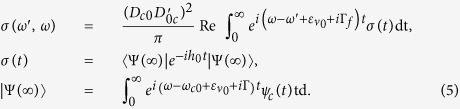


Here, *ω (ω*′) and **e** (**e**') are the frequency and polarisation vector of incoming (outgoing) photon; 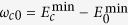 is the energy between the PES minima of the ground and core-excited states; 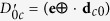, *D*_*c*0_ = (**e **· **d**_*c*0_), **d**_*c*0_ is the absorption transition dipole moment; Γ and Γ_*f*_ are the core-hole and final state lifetime broadening, respectively; *h*_0_ end *h*_*c*_ are defined in Methods Eq. (17).

### RIXS at 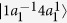 resonance

**Figure 3:**
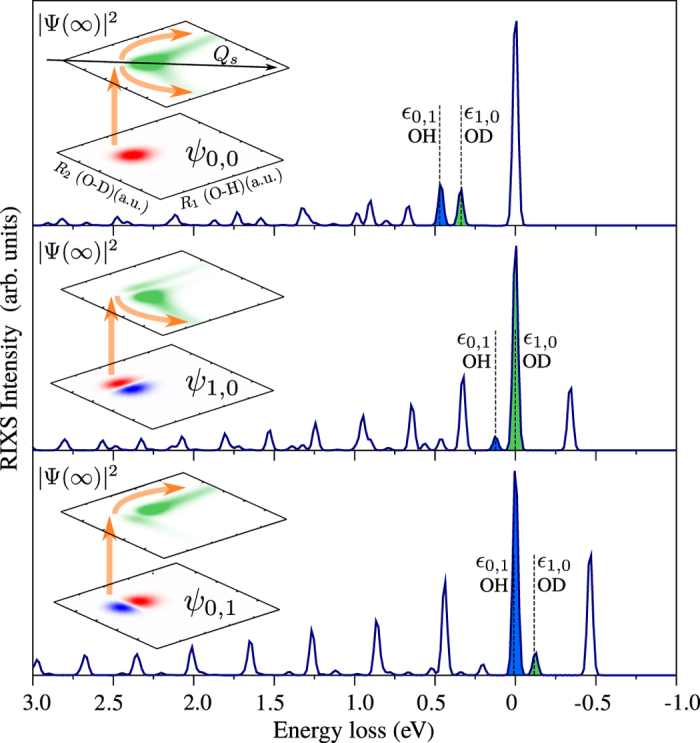
RIXS spectra at 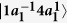 resonance of vibrationally excited HDO molecule. The left-hand side shows the shape of the initial vibrational wave function in the ground state and the core-excited wave packet |Ψ(∞)|^2^ (5). The *ε*_1,0_ and *ε*_0,1_ dashed lines represent the energy position of the *ψ*_1,0_ and *ψ*_0,1_ final vibrational states, respectively. The photon frequency *ω* is tuned in resonance with the top of the X-ray absorption profile (see also Supplementary Fig. S2).

To show the strong sensitivity of the studied effect to the core-excited state character, we first analyse RIXS via the dissociative 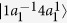 core-excited state, where the fragmentation of HDO along the O-H and O-D bonds is strongly asymmetric. Conventional RIXS starts from the lowest vibrational state *ψ*_0,0_ which is delocalised (see Fig. 3, upper panel). This delocalisation is preserved in the core-excited state, as one can see from the spatial distribution of the integral wave packet |Ψ(∞)〉 (5), except for an asymmetry between the O-H and O-D dissociation channels. The O-H branch of |Ψ(∞)|^2^ is more pronounced than the O-D branch due to slower dissociation of the twice as heavy deuterium atom. The delocalisation is reflected in the RIXS spectrum (Fig. 3, upper panel), which is defined by the overlap between the core-excited wave packet |Ψ(∞)〉 and the final vibrational wave function^26^ (Supplementary Eq. (S1)). Due to this fact, the final vibrational states *ψ*_1,0_ and *ψ*_0,1_ (Fig. 3, left) have almost the same intensities in the RIXS spectrum (peaks *ε*_1,0_ and *ε*_0,1_ in Fig. 3, respectively). The reason for the slightly weaker intensity of the *ψ*_1,0_ (O-D) resonance is the above mentioned slower dissociation of deuterated O-D bond in relation to the O-H bond.

The picture changes qualitatively when the RIXS starts from the excited vibrational state *ψ*_1,0_ (*ψ*_0,1_), localised on the O-D (O-H) bond (Fig. 2), resulting in a clear bond selectivity of the RIXS (Fig. 3, mid and bottom panels). Indeed, when the RIXS starts from *ψ*_1,0_ (Fig. 3, mid panel), the intensity of the O-D peak (*ε*_1,0_) is much larger than the intensity of the *ε*_0,1_ resonance. This phenomenon is qualitatively illustrated by the scheme on the left hand-side of Fig. 3 (see also Fig. 1). The complementary scenario was observed for RIXS starting from *ψ*_0,1_ (Fig. 3, lower panel). In this case, the *ε*_0,1_ RIXS resonance, which corresponds to O-H bond, has the strongest intensity.

### RIXS at the 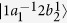 resonance

The bound 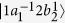 PES exhibits a “canyon-like” shape along the symmetric stretching coordinate *R*_1_ = *R*_2_ (Fig. 1). Even though the ground state wave functions of HDO are completely localised, the core-excited wave functions are very similar to the H_2_O molecule (Fig. 2), as discussed above. In order to have a complete picture of the localisation in the IR-pumped RIXS via the 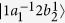 resonance, we consider for each initial vibrational state four different incoming photon frequencies tuned in resonance with the 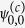, 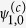, 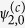 and 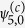 core-excited vibrational states of HDO (Fig. 2 and Supplementary Fig. S2). The resonant condition is described by the detuning from the excitation energy of the lowest core-excited vibrational level 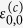 as


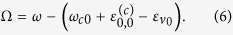


**Figure 4:**
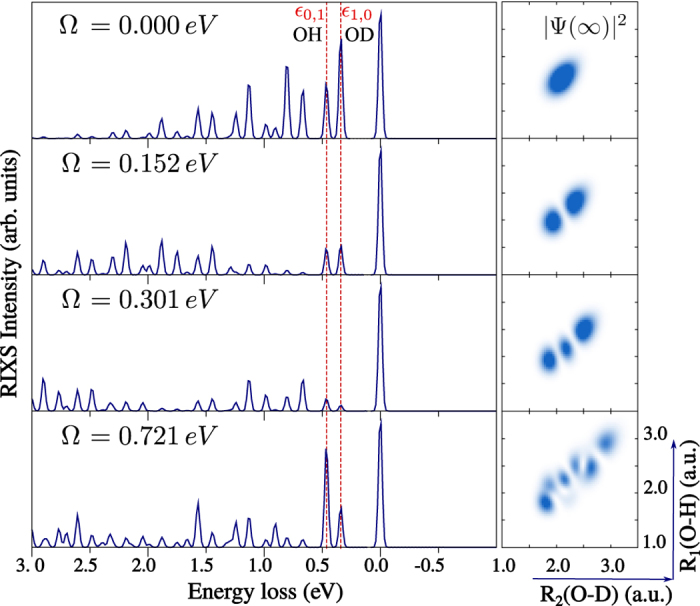
HDO RIXS spectra via 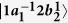 resonance for detuning Ω = 0.000, 0.152, 0.301, 0.721 eV, corresponding to the excitation in resonance with the vibrational states 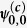, 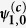, 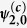, 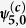 of the core-excited state (see Fig. 2 and Supplementary Fig. S2). The initial vibrational state *ψ*_0,0_ is used (no IR-pump). At the right-hand side, we show the squared core-excited wave packet |Ψ(∞)|^2^ (5)) for each detuning.

Let us consider the conventional RIXS from the lowest vibrational state *ψ*_0,0_ (Fig. 4). The delocalisation of the vibrational state *ψ*_0,0_ is preserved in the core-excited state (similar to the 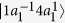 case) and the core-excited wave packet is distributed over the both bonds *R*_2_(*D* − *O*) and *R*_1_(*O* − *H*) for all considered detuning values (Fig. 4, right-hand side). One can clearly see this delocalisation in the RIXS spectra (left panels in Fig. 4), which have comparable intensities of the final vibrational states *ψ*_1,0_ and *ψ*_0,1_ localised on O-D and O-H bonds, respectively (peaks *ε*_1,0_ and *ε*_0,1_ in Fig. 4). Thus, when a delocalised core-excited wave packet is created, the selectivity of the final state vibrations localised on the O-H or O-D bonds is lost. The dependence of the relative intensity of the *ε*_1,0_ and *ε*_0,1_ peaks on detuning stems from the change of the core-excited wave packet shape with excitation energy (see Supplementary Notes 3).

**Figure 5:**
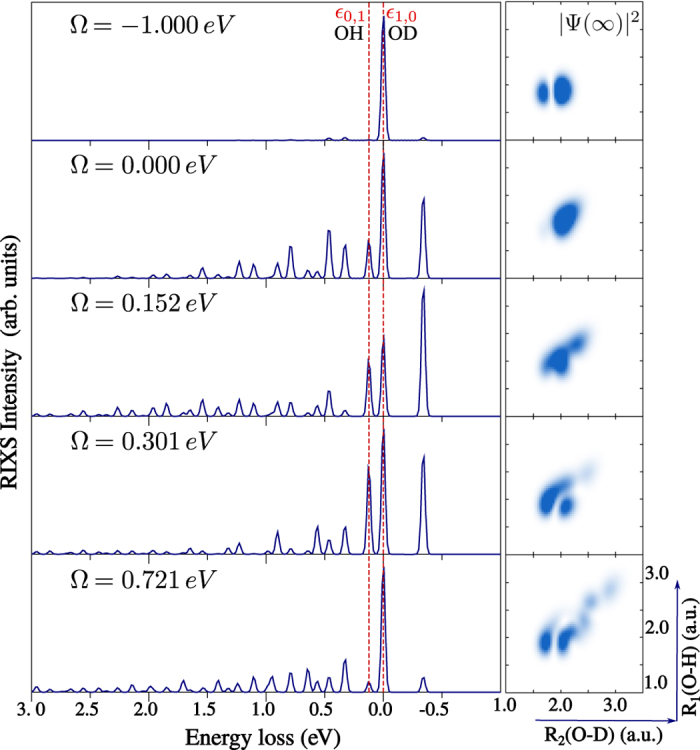
HDO RIXS spectra and core-excited wave packets at the 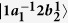 resonance. The molecule is initially IR-pumped to the *ψ*_1,0_ vibrational state. Notations are the same as in Fig. 4.

**Figure 6:**
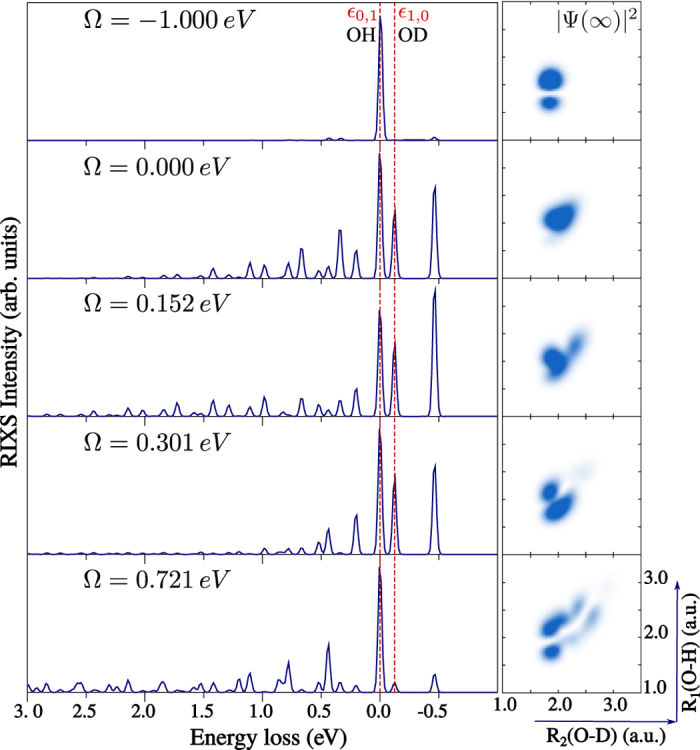
HDO RIXS spectra and core-excited wave packets at the 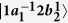 resonance. The molecule is initially IR-pumped to the *ψ*_0,1_ vibrational state. Notations are the same as in Fig. 4.

Let us now focus on the 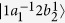 RIXS from one of the localised initial vibrational state *ψ*_1,0_ (Fig. 2). Contrary to the case of the 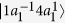 core-excited state, a delocalised core-excited wave packet is now formed for detuning Ω = 0.000 and 0.152 eV (Fig. 5, right-hand side), due to the “canyon-like” shape of the 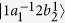 PES along the symmetric stretching coordinate (see Fig. 1). The tight confinement of the nuclear motion in this PES forces the H and D atoms to oscillate coherently along the symmetric stretching coordinate, like in the H_2_O molecule, in spite of the large mass difference between the H and D atoms. This effect is clearly reflected in the RIXS spectrum, as the intensities of the peaks *ε*_1,0_ and *ε*_0,1_ are comparable for low positive detuning energies (Fig. 5, Ω = 0.152, 0.301 eV) and no bond selectivity is observed. The same behaviour is observed for RIXS initiated from the excited vibrational state *ψ*_0,1_ localised on the O-H bond (Fig. 6). As one can see from the topmost panel of Figs 5 and 6 (Ω = −1.00 eV), the core-excited wave packet is fully localised along the bond, exactly as the vibrational wave function initial to the RIXS process. The case of Ω = 0.0, which shows smaller intensity of OH peak, corresponds to a transition state between the fully localised (Ω = −1.0 eV) and almost fully delocalised (Ω = 0.152 eV) cases. This phenomenon is related to the scattering duration and the collapse of the wave function, which will be described in the following section.

The delocalisation of the core-excited wave packet on the 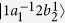 PES is broken for higher excitation energy (Ω ≥ 0.301 eV), as one can see from Figs 5 and 6. This happens due to the anharmonicity of the the 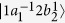 PES which makes the spacing between levels smaller as we reach higher vibrational excitations. When the spacing between the vibrational levels becomes comparable or smaller than the core-hole lifetime broadening (Γ = 0.08 eV), the core-excited wave packet becomes a mixture of several core-excited vibrational states. This coherent superposition of the core-excited states leads to a loss of delocalisation of the vibrational wave function, and thus retains localisation of the ground state nuclear dynamics along the bonds. Analysing the RIXS spectrum at Ω = 0.301 eV (Figs 5 and 6), one can see that core-excited wave packet shows a localisation tendency, but that it is still insufficient to drive the bond selectivity of the final states in RIXS so that the *ψ*_1,0_ and *ψ*_0,1_ peaks have comparable intensities. The localisation of the core-excited wave packet is more pronounced for higher excitation energy Ω = 0.721 eV, manifested as a strong asymmetry of the RIXS intensities *ε*_1,0_ and *ε*_0,1_ (see Figs 5 and 6).

### Dynamics of wave function collapse

Different orientations of the initial vibrational state *ψ*_1,0_ localised along the OD bond with respect to the eigenstate of *h*_*c*_ delocalised between the OH and OD bonds (Fig. 1) give an interesting opportunity to visualise the gradual rotation of *ψ*_1,0_ in the direction of the valley in the 2*b*_2_ core-excited state potential in the course of X-ray absorption or RIXS measurements. Such a rotation has direct relation to the cornerstone quantum mechanical problem - the collapse of the wave function. Indeed, one of the most debated postulates of quantum mechanics is the collapse of a quantum system from a coherent superposition of several quantum states to a single one at the moment of a measurement. The wave function collapse, often regarded as a sudden and indeterministic process during a measurement, is related to von Neumann’s projection postulate, and remains one of most controversial magenta aspects of quantum physics^30^,^31^.

Let us remind the expression for the absorption cross section of continuous wave (CW) light in the vicinity of the resonance with the *ν*_*c*_ vibrational level of a core-excited state with the energy 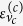, selected by the resonant condition:


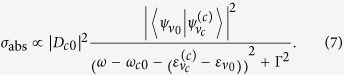


The initial *ν*_0_ vibrational level of the ground electronic state is not an eigenstate of the core-excited Hamiltonian, and can thus be written as a coherent superposition of vibrational states *ν*_*c*_ of the core-excited state


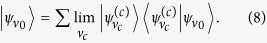


According to (7), the absorption probability is defined by the projection of the initial wave packet (8) on a single eigenstate 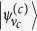 of the core-excited state. This leads to an erroneous conclusion that there is a sudden collapse from the coherent superposition (8) to a single state. However, Eq. (7) assumes that the duration of the measurement is much longer than the lifetime of the core-excited state 1/Γ, defined mainly by the Auger decay duration in our case. To describe properly the wave function collapse, one has to take into account the finite time of the measurement^32^.

The HDO system, investigated above, is a good example to demonstrate the gradual Schrödinger evolution of the quantum system to a single “collapsed” state during the process of measurement. To describe the dynamics of the wave function collapse, one has to include in the formalism the interaction of the quantum system with the spectral recording device. Let us consider the dynamics of the absorption of low intense X-ray light 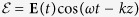 propagating along the *z* axis. Here **E**(*t*) = **e***E*(*t*) and *k* = *ω*/*c*. The X-ray field, resonant to the transition frequency, creates a coherent superposition of the ground |*ϕ*_0_〉 and core-excited |*ϕ*_*c*_〉 electronic states (see Supplementary Notes 1,2)


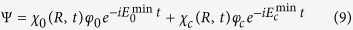


and the polarisation 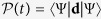. The evolution of the nuclear wave packet *χ*_*c*_(*R, t*) in the core-excited state is non-unitary and obeys the Schrödinger equation with the damping


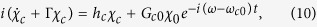


where 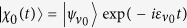, **d**_*c*0_ = 〈*ϕ*_0_|**d**|*ϕ*_*c*_〉 is the electronic transition dipole moment, 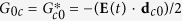 is the Rabi frequency. The light-induced polarisation changes the intensity of the field, described by the wave equation within the slowly varying amplitude approximation as (see Supplementary Eq. (S9))


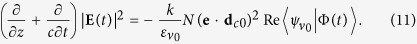


This equation makes the direct link between the absorption coefficient at the time *t* and the field-dependent wave packet |Φ(*t*)〉


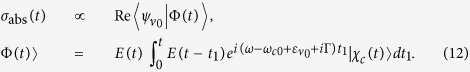


Let us consider a rectangular pulse with the duration *T*. In this case the wave packet


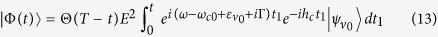


gradually converges to the wave packet |Φ(∞)〉 (5) for the CW X-ray field, when the interaction time *t* → ∞ (*T* = ∞). Here Θ(*T* − *t*) is the step function.

**Figure 7:**
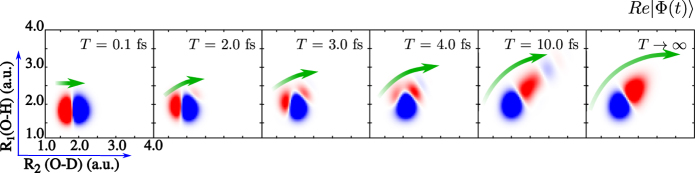
Dynamics of the wave function collapse in the 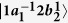 core-excited state of HDO, shown through the nuclear wave packet |Φ(*T*)〉 for different pulse duration *T*.

Considering an increasing pulse duration *T*, one can observe the rotation of the nuclear wave packet (Fig. 7), which is nothing else than the gradual evolution of the initial state 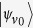 to the eigenstate state of the nuclear Hamiltonian of core-excited state. In the present case, the initial |*ψ*_1,0_〉 wave function, aligned along the O-D bond, transforms into the eigenstate 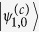 of the core-excited state nuclear Hamiltonian (“collapsed state”), aligned along the valley of the core-excited potential. Thus, the wave packet |Φ(*t*)〉 approaches the collapsed state evolutionary, according to the Shrödinger equation with the damping Γ, see Eq. (10).

There is an alternative way to observe the dynamics of the wave function collapse using the stationary RIXS measurement with CW X rays. Indeed, the wave packet in the core-excited state (5) can be written in the following form (see Supplementary Notes)


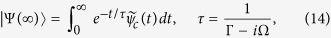


where 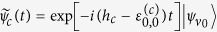. The complex time *τ* has the physical meaning of a scattering duration^33^, defined by two characteristic time values. The first one is the lifetime of the core-excited state 1/Γ, which is responsible for its irreversible decay. The second one 1/|Ω|, originating from the detuning Ω, can be associated with a dephasing time because the contributions to the integral in Eq. (14) interfere destructively owing to the phase difference Ω(*t*_2_ − *t*_1_). The destructive interference quenches the contributions at times *t* > *τ*_*s*_


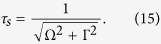


**Figure 8:**
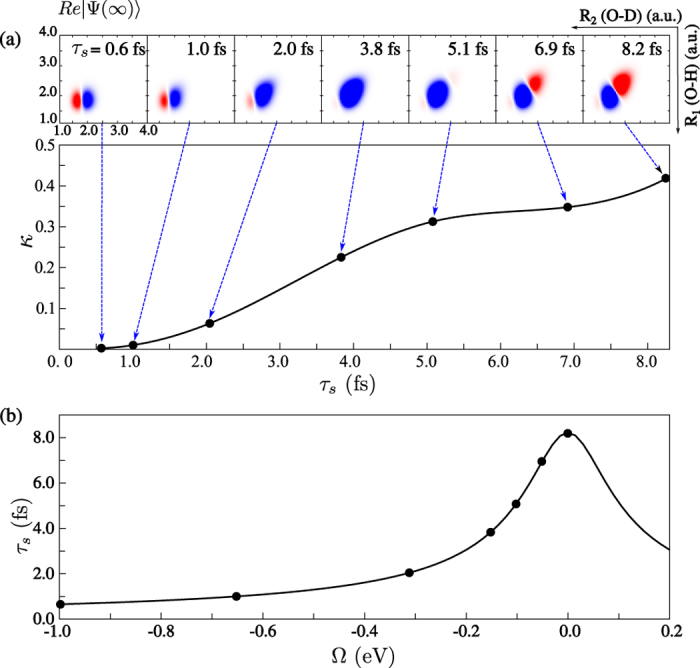
Dynamics of the collapse of the wave function pictured by the scattering duration *τ*_*s*_ (15). (**a**) Degree of the delocalisation *κ* (16) versus scattering duration *τ*_*s*_. The top panel shows the real part of the core-excited wave packet |Ψ(∞)〉 for the different values of the scattering duration *τ*_*s*_. (**b**) Scattering duration as a function of detuning defined here with respect to core-excitation to the 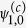 vibration state, 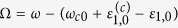.

Now we are able to see the gradual evolution of the wave packet |Ψ(∞)〉 from *τ*_*s*_ ≈ 0 (large detuning) to the region of large *τ*_*s*_ = 1/Γ = 8.23 fs at strict resonance (Fig. 8a). The advantage of this technique is that we can visualise the Schrödinger evolution using the RIXS spectra, as it is depicted on Fig. 8a by the relative intensity of the O-H peak


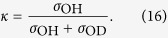


Here, *σ*_O*H*_ and *σ*_O*D*_ denote the *ε*_0,1_ and *ε*_1,0_ peak intensities, respectively, in the 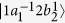 RIXS spectra with initial vibrational state *ψ*_1,0_ (Fig. 5). When the scattering duration is short (large |Ω|), the shape of the wave packet |Ψ(∞)〉 is close to the initial wave function *ψ*_1,0_ localised on the O-D bond and only the resonance related to the O-D stretching is observed in the RIXS spectrum (Fig. 5, top panel). When the scattering duration is sufficiently long (small Ω), the wave packet |Ψ(∞)〉 has time to align along the valley of the 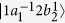 PES, so its shape becomes similar to the core-excited state eigenstate 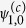. One can see that the evolution of the wave packet |Ψ(∞)〉 in the scattering duration time (Fig. 8a) is rather similar to the evolution of the wave packet |Φ(*t*)〉 (Fig. 7). The comparison between the time evolution of the wave packet |Ψ(∞)〉 and the relative intensity of the O-H resonance (Fig. 8a) demonstrates the possibility to observe the dynamics of the wave function collapse by changing the scattering duration, controlled by the detuning in the RIXS experiment (Fig. 8b). We note, that our simulations do not include the close-lying core-excited state 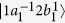 (0.8 eV above the 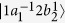 state)^26^. Due to this circumstance, we exclude in Fig. 8 the region Ω > 0.2 eV, where interference between the 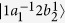 and 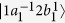 RIXS channels becomes important.

## Conclusion

In the present work we have demonstrated how a gradual collapse, or localisation, of nuclear wave functions of a triatomic system, HDO, can be regulated by frequency detuning of X-ray scattering experiment. This is shown by a theoretical analysis of quasi-elastic IR-pump X-ray-probe RIXS spectra of the HDO molecule selectively excited to a few localised vibrational levels in the ground electronic state. The localisation/delocalisation problem in the core-excited states was studied by the time-dependent wave-packet propagation method. It is shown that the localisation of the ground vibrational state is preserved in the course of the core-excitation in the dissociative 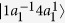 state, while in the case of the bound 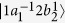 core-excited state the nuclear wave packet is trapped in the valley of the PES aligned between the O-H and O-D bonds and localisation is broken. In this state, the narrow “canyon-like” symmetric potential overcomes the “localising” role of the kinetic energy operator, and the nuclear wave packet is confined along the valley of the potential. We show that the degree of the delocalisation in the 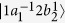 state decreases when the probe X-ray photon is tuned in resonance with higher vibrational levels of the core-excited state (Ω ≥ 0.300 eV). The studied isotopomer HDO is thus found to be a good showcase system to examine the gradual evolution of the nuclear wave function to a single eigenstate of the nuclear Hamiltonian in a core-excited state, thus featuring a gradual rather than instantaneous collapse. We described two schemes of possible experimental observation of the collapse phenomena – using short X-ray free-electron laser pulses of controlled duration and by controlling the detuning from the resonance using a CW X-ray synchrotron source. The possibility of using the pump-probe RIXS technique to dynamically control the degree of delocalisation of vibrations, here demonstrated for HDO, can also be applied to other asymmetric triatomic systems.

## Methods

The PESs of the ground and 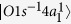 and 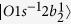 core-excited states were computed with the MOLCAS 8.0 package^34^ using the scalar-relativistic restricted-active-space self-consistent-field (RASSCF) method^35^ followed by second-order perturbation theory (RASPT2) method^36^, with the ANO-RCC^37^ basis set. Details of the RASPT2 calculations can be found in our previous study of H_2_O^26^. All wave packet simulations were performed employing the eSPec program^38^ using nuclear Hamiltonian


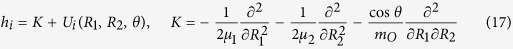


written in the valence coordinate representation^39^. Here, the label *i* = 0, *c* denotes the ground and core-excited electronic states, respectively, *R*_1_ and *R*_2_ are the lengths of the O-H and O-D bonds, the bond angle *θ* is here assumed constant in our model and equal to the equilibrium geometry of H_2_O *θ* = 104.21° 26,27, 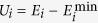 is the potential energy with respect to the bottom 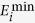 of the potential energy surface *E*_*i*_(*R*_1_, *R*_2_).

## Additional Information

**How to cite this article**: Ignatova, N. *et al*. Gradual collapse of nuclear wave functions regulated by frequency tuned X-ray scattering. *Sci. Rep.* **7**, 43891; doi: 10.1038/srep43891 (2017).

**Publisher's note:** Springer Nature remains neutral with regard to jurisdictional claims in published maps and institutional affiliations.

## Supplementary Material

Supplementary Information
