# Supplementary material for: Gradual collapse of nuclear wave functions regulated by frequency tuned X-ray scattering
Source: Sci Rep. 2017 Mar 7;7:43891. doi: 10.1038/srep43891 (PMC5339714; doi:10.1038/srep43891)
Supplement: Supplementary Information [file srep43891-s1.pdf]

SUPPLEMENTARY INFORMATION

Gradual collapse of nuclear wave functions regulated by frequency tuned X-ray scattering

by Nina Ignatova, Vinícius V. Cruz, Rafael C. Couto, Emelie Ertan, Andrey Zimin, Freddy F. Guimarães, Sergey Polyutov, Hans Ågren, Victor Kimberg, Michael Odelius, and Faris Gel'mukhanov.

Supplementary Notes 1. Time-dependent representation for XAS and RIXS cross-sections

The RIXS cross section is given by the Kramers-Heisenberg equation

$$\sigma(\omega',\omega) = (D_{c0}D'_{0c})^2 \sum_{\nu_f} |F_{\nu_f}|^2 \Delta(\omega-\omega'-\varepsilon_{\nu_f}+\varepsilon_{\nu_0},\Gamma_f),$$

$$F_{\nu_f} = -\imath\langle \nu_f|\Psi(\infty)\rangle, \tag{S1}$$

where the scattering amplitude F_{ν_f} is defined by the projection of a coherent superposition of core-excited vibrational states

$$|\Psi(\infty)\rangle = \imath \sum_{\nu_c} \frac{|\nu_c\rangle\langle\nu_c|\nu_0\rangle}{\omega-\omega_{c0}+\varepsilon_{\nu_0}-\varepsilon^{(c)}_{\nu_c}+\imath\Gamma} \tag{S2}$$

on the final vibrational state $|\nu_f\rangle$. Here ω and ω' are the incoming and outgoing photon frequency, respectively; index $\nu = (n_s, n_a)$ or $\nu = (n_D, n_H)$ labels the 2D vibrational state, $D'_{c0} = (\mathbf{e}'\cdot\mathbf{d}_{c0})$, $D_{c0} = (\mathbf{e}\cdot\mathbf{d}_{c0})$, $\mathbf{d}_{c0}$ is the absorption transition dipole moment; $\mathbf{e}$ and $\mathbf{e}'$ are the polarisation vectors of the incoming and outgoing photons, respectively; $\Delta(x,\Gamma) = \Gamma/\pi(x^2+\Gamma^2)$; $\omega_{c0} = E_c(R^{(c)}_0) - E_0(R_0)$ is the transition energy of the adiabatic transition from the bottom of ground state PES to the bottom of core-excited PES ($R^{(c)}_0$ is the position of the minimum of core-excited PES); ε_{ν_0}, $\varepsilon^{(c)}_{\nu_c}$ and ε_{ν_f} are the vibrational energy of the ground, core-excited and final state, respectively; Γ and Γ_f are the core-hole and final state lifetime broadening, respectively.

Our numerical simulations are based on the wave packet technique. Therefore we use the time-dependent representation for the RIXS (Eq.S1) cross-section. The X-ray absorption (XAS) cross section [1]

$$\sigma_{\text{abs}}(\omega) = D^2_{c0}\sum_{\nu_c}|\langle\nu_c|\nu_0\rangle|^2\Delta(\omega-\omega_{c0}-\varepsilon^{(c)}_{\nu_c}+\varepsilon_{\nu_0},\Gamma^2) = D^2_{c0}\text{Re}\langle\nu_0|\Psi(\infty)\rangle \tag{S3}$$

can be written as projection of the integrated core-excited wave packet $|\Psi(\infty)\rangle$ on the initial vibrational state $|\nu_0\rangle$ of the ground electronic state. Both XAS and RIXS processes are sensitive to the nuclear dynamics in core-excited state defined by the nuclear wave packet

$$|\psi_c(t)\rangle = e^{-\imath h_c t}|\nu_0\rangle, \tag{S4}$$

as one can see from the time-dependent representation for the XAS cross section

$$\sigma_{\text{abs}}(\omega) = -\frac{D^2_{c0}}{\pi}\text{Re}\int_0^\infty e^{\imath(\omega-\omega_{c0}+\varepsilon_{\nu_0}+\imath\Gamma_f)t}\sigma(t)dt,$$

$$\sigma_c(t) = \langle\nu_0|\psi_c(t)\rangle \tag{S5}$$

and RIXS cross section

$$\sigma(\omega',\omega) = \frac{(D_{c0}D'_{0c})^2}{\pi}\text{Re}\int_0^\infty e^{\imath(\omega-\omega'+\varepsilon_{\nu_0}-\Gamma t}\sigma(t)dt,$$

$$\sigma(t) = \langle\Psi(\infty)|e^{-\imath h_0 t}|\Psi(\infty)\rangle, \tag{S6}$$

$$|\Psi(\infty)\rangle = \int_0^\infty e^{\imath(\omega-\omega_{c0}+\varepsilon_{\nu_0}+\imath\Gamma)t}\psi_c(t)dt.$$

One should mention here that the decay rate of the core-excited state Γ plays a crucial role in the nonunitary evolution of $|\Psi(\infty)\rangle$. Indeed, equation

$$|\Psi(\infty)\rangle = \lim_{T\to\infty}|\Psi(T)\rangle, \tag{S7}$$

$$|\Psi(T)\rangle = \imath\sum_{\nu_c}\frac{|\psi^{(c)}_{\nu_c}\rangle\langle\psi^{(c)}_{\nu_c}|\psi_{\nu_0}\rangle\left(1-e^{\imath(\omega-\omega_{c0}+\varepsilon_{\nu_0}-\varepsilon^{(c)}_{\nu_c})T}e^{-\Gamma T}\right)}{\omega-\omega_{c0}+\varepsilon_{\nu_0}-\varepsilon^{(c)}_{\nu_c}+\imath\Gamma}$$

shows that one can reach a single core-excited state $\psi^{(c)}_{nu_c}$ at the resonance only when the measurement time is longer than the lifetime $T > 1/\Gamma$.

Supplementary Notes 2. Interaction of molecule with the X-ray pulse

The light-induced polarisation

$$\mathscr{P}(t) \approx \sum_{n,m=0,c} \mathbf{d}_{nm} \langle \chi_n(R,t) | \chi_m(R,t) \rangle e^{\imath (E_n^{\min} - E_m^{\min}) t}. \tag{S8}$$

changes the intensity of the X-ray field described by the wave equation within the slowly varying amplitude approximation as

$$\begin{aligned} \left(\frac{\partial}{\partial z} + \frac{\partial}{c \partial t} \right) |\mathbf{E}(t)|^2 &= -\mathrm{Im} \left(\frac{2kN}{\varepsilon_{v_0}} (\mathbf{E}^*(t) \cdot \mathscr{P}(t)) e^{\imath(\omega - \omega_{c0})t} e^{\imath \varepsilon_{v_0} t} \right) \\ &= -\mathrm{Im} \left\{ \frac{2k}{\varepsilon_{v_0}} N (\mathbf{E}^*(t) \cdot \mathbf{d}_{c0}) \langle \psi_{v_0} | \chi_c(t) \rangle e^{\imath(\omega - \omega_{c0} + \varepsilon_{v_0})t} \right\}, \end{aligned} \tag{S9}$$

where the right-hand side describes the absorption coefficient, N is the concentration of molecules.

Supplementary Notes 3. Vibrational states of ground and core-excited electronic states of H_2O and HDO

The ground and core-excited electronic states' vibrational wave functions of H_2O and HDO, discussed in the paper (see Fig.2 and Fig. S1), were computed using the 2D time-independent Schrödinger equation by direct signalisation of the Hamiltonian.

We here comment on the variation of the relative intensity of OD and OH peaks with the detuning (Fig. 4), e.g. inverted relative intensities $\Omega = 0.0$ and 0.721 eV. The reason for this is strong dependence of the core-excited wave packet on the detuning. Indeed, the wave packet copies the shape the core-excited vibrational state $\psi^{(c)}_{0,0}$, when the photon energy is tuned in resonance with this state ($\Omega = 0.0$). The size a_{OD} of $\psi_{0,1}$ along the OD bond is smaller than the size a_{OH} of $\psi_{1,0}$ along the OH bond ($a_{OD}/a_{OH} = \sqrt{\omega_{OD}/\omega_{OH}} \approx 0.86$) due to the smaller OD vibrational frequency (see Fig. S1). This makes the overlap of $|\Psi(\infty)|^2$ with $\psi_{0,1}$ smaller than with $\psi_{1,0}$, which explains the smaller intensity of the OH peak in comparison with the OD resonance (see Fig. 4).

In contrast to $\Omega = 0.0$, the shape of the wave packet for higher detuning $\Omega = 0.721$ eV is strongly affected by the interference of two close lying vibrational states $\psi^{(c)}_{2,1}$ and $\psi^{(c)}_{5,0}$ (see Fig. S1) and becomes closer to the shape of the wave function $\psi_{0,1}$ than to the shape of the wave function $\psi_{1,0}$ in the region of localisation of these wave functions ($R < 2.0$ a.u.). This results in increase of the intensity of the OH peak.

Supplementary Notes 4. X-ray absorption spectrum of HDO

In the RIXS at $|1a_1^{-1} 2b_2^1\rangle$ state, the excitation energy was tuned in resonance with several core-excited vibrational levels, as demonstrated in Fig. S2. For convenience, we introduced the detuning Ω (see Eq.(6)) with respect to the excitation from ψ_{v_0} to the lowest vibrational level of the core-excited state $\psi^{(c)}_{0,0}$.

Supplementary Figures

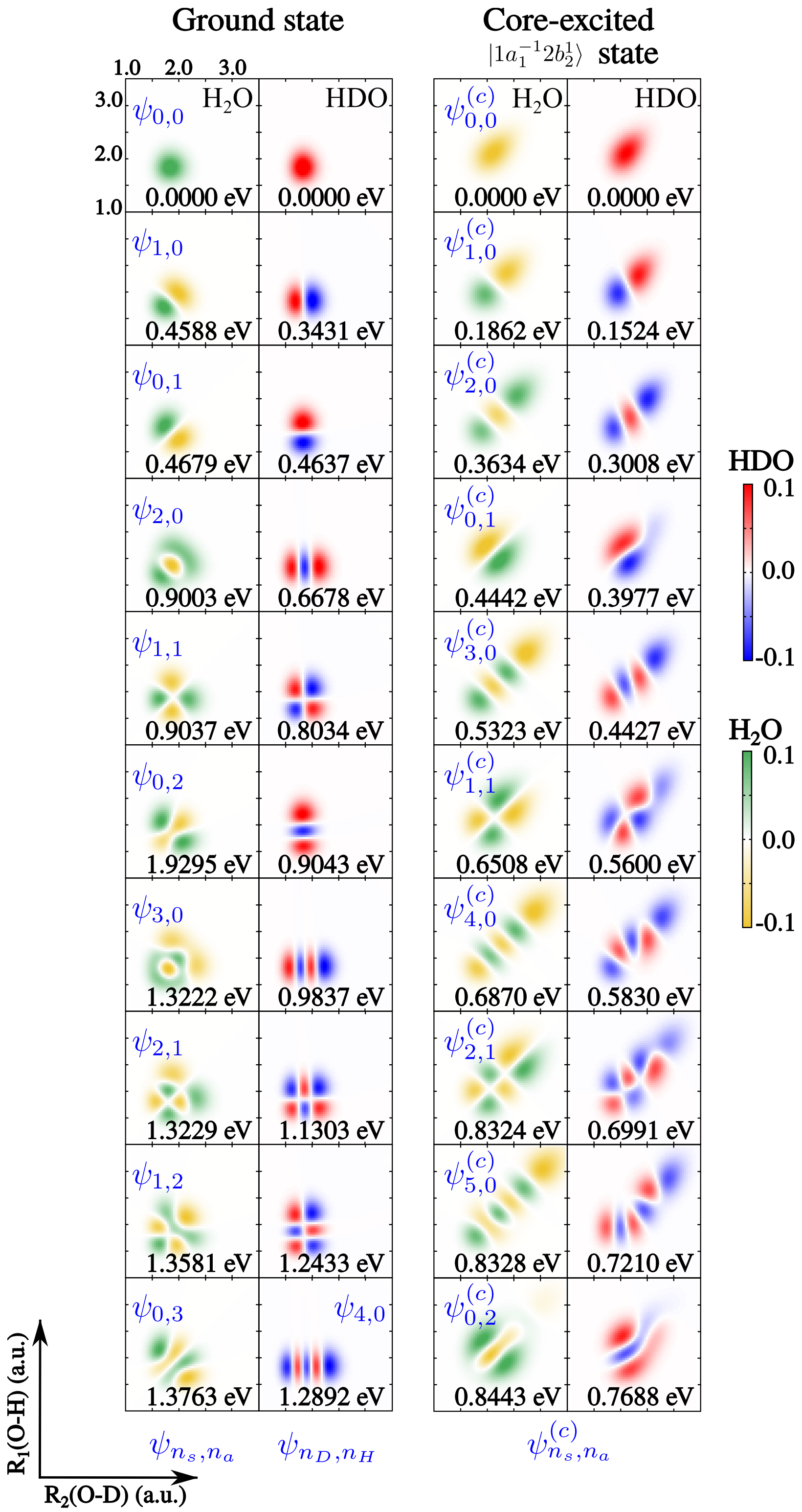

Supplementary Figure S1. Vibrational wave functions and eigenvalues for the ground and core-excited $|1a_1^{-1}2b_2^1\rangle$ states of H_2O and HDO. The number inside each subpanel represents the energy of vibrational state with respect to zero-point energy.

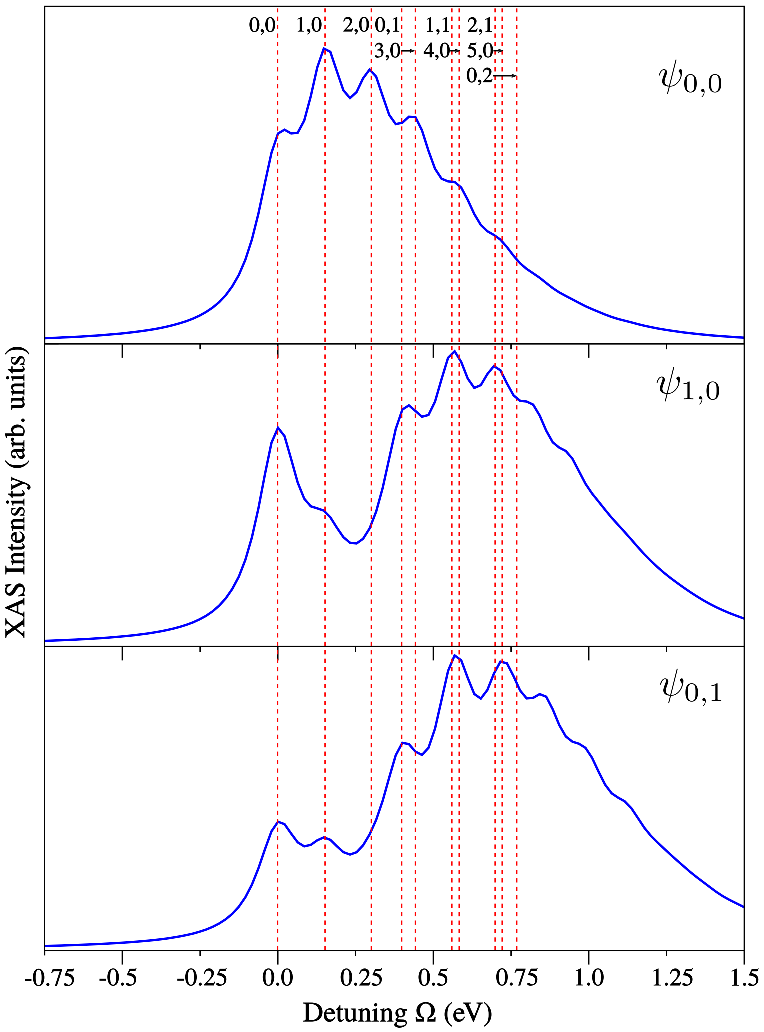

Supplementary Figure S2. Simulated $|1a_1^{-1}2b_2^1\rangle$ X-ray absorption spectrum of HDO for different initial vibrational states. The dashed lines indicates the position of the vibrational levels of the $|1a_1^{-1}2b_2^1\rangle$ core-excited state.

Supplementary References

1. Gel'mukhanov, F. & Ågren, H. Resonant X-ray Raman scattering. *Phys. Rep.* **312**, 87–330 (1999).
